# The Role of Adjuvant Radiotherapy for the Treatment of Resected High-Risk Stage III Cutaneous Melanoma in the Era of Modern Systemic Therapies

**DOI:** 10.3390/cancers15245867

**Published:** 2023-12-16

**Authors:** Seth Kibel, Nathan Kuehne, Mauricio Fernando Ribeiro, Thiago P. Muniz, Xiang Y. Ye, Anna Spreafico, Samuel D. Saibil, Alexander Sun, David Y. Mak, Diana Gray, Bailie Jones, Philip Wong, Marcus O. Butler

**Affiliations:** 1 Department of Medicine, Temerty Faculty of Medicine, University of Toronto, Toronto, ON M5S 3H2, Canada; seth.kibel@mail.utoronto.ca (S.K.); nathan.kuehne@mail.utoronto.ca (N.K.);; 2 Division of Medical Oncology and Hematology, Princess Margaret Cancer Centre, University Health Network, Toronto, ON M5G 2M9, Canada; 3 Department of Biostatistics, Princess Margaret Cancer Centre, University Health Network, Toronto, ON M5G 2M9, Canada; 4 Radiation Medicine Program, Princess Margaret Cancer Centre, University Health Network, Toronto, ON M5G 2M9, Canada; 5 Department of Immunology, University of Toronto, Toronto, ON M5S 3H2, Canada

**Keywords:** adjuvant therapy, melanoma, immunotherapy, radiotherapy, locoregional recurrence

## Abstract

**Simple Summary:**

Historically, patients undergoing complete lymphadenectomy (CLD) for clinically evident nodal disease were candidates to receive adjuvant radiotherapy (RT), with the goal of reducing the risk of lymph node basin (LNB) relapse. However, most recent systemic therapy (ST) trials investigating adjuvant immune checkpoint inhibitors and targeted therapies have excluded patients who had received adjuvant RT prior to ST. Therefore, the role of this therapy is under-investigated, and patients who may have previously received adjuvant RT may now be receiving adjuvant ST and forgoing adjuvant RT. We observed that there was a significant shift away from the use of radiotherapy toward systemic therapies after 2015 compared to before 2015 in a population that met indications for radiotherapy. We further found that the LNB recurrence rate was similar between those treated with adjuvant RT and ST, and ST was associated with a reduced incidence of any recurrence or progression compared to adjuvant RT.

**Abstract:**

Modern adjuvant systemic therapies (STs) have revolutionized the management of stage III melanoma. Currently, the role of adjuvant radiotherapy (RT) remains unclear. In this single-center retrospective study, patients with clinically detectable stage III melanoma with high-risk features for lymph node basin (LNB) recurrence and whose tumors were fully resected with complete lymphadenectomy (CLD) between 2010 and 2019 were assessed. We determined the cumulative incidence (CIF) of LNB recurrence and any disease recurrence or progression using competing risk analysis. A total of 108 patients were identified; the median age was 59 years (24–92), and 74 (69%) were men. A total of 51 (42%) received adjuvant RT, 22 (20%) received adjuvant ST, and 35 (32%) received no adjuvant therapy. The advent of ST changed clinical practice, with a significant increase in the use of adjuvant ST and a decrease in the use of RT when comparing practice patterns before and after 2015 (*p* < 0.001). The 3-year CIF of LNB recurrence was similar in patients treated with adjuvant RT (6.3%) and adjuvant ST (9.8%). The 3-year CIF of any disease recurrence or progression was lower in patients receiving adjuvant ST (24%) compared to those receiving adjuvant RT (52%) or no adjuvant therapy (55%, *p* = 0.06). Three-year overall survival (OS) was not significantly different in patients treated with ST compared to those not treated with any ST (*p* = 0.118). Despite ST replacing RT as the dominant adjuvant treatment modality, this change in practice has not resulted in increased LNB recurrence for patients at high risk of LNB recurrence following CLD.

## 1. Introduction

Immune checkpoint inhibitors (ICIs) and BRAF/MEK targeted therapies have recently revolutionized the treatment landscape for melanoma. While initial evidence supported the use of these therapies in the metastatic setting, recent studies have extended their indication to resected, locally advanced patients as well [1,2,3,4,5,6,7]. Stage III melanoma encompasses a heterogeneous group of patients with 5-year overall survival ranging from 93% in patients with stage IIIA disease to 32% in patients with stage IIID [8], demonstrating that patients with clinically evident lymph node involvement have reduced overall survival when compared to those presenting with clinically occult nodal disease [9]. Previously, patients undergoing complete lymphadenectomy (CLD) for clinically evident nodal disease were candidates to receive adjuvant radiotherapy (RT), with the goal of reducing the risk of lymph node basin (LNB) relapse [10,11,12]. In Ontario, Canada, these criteria were incorporated into the 2016 Cancer Care Ontario (CCO) guidelines for the use of adjuvant radiation therapy in resected, locally advanced cutaneous melanoma patients [13].

However, most recent systemic therapy (ST) trials investigating adjuvant ICIs and targeted therapies have excluded patients who had received adjuvant RT prior to ST [5,14,15,16]. Therefore, the role of this therapy is underinvestigated, and patients who may have previously received adjuvant RT may now be receiving adjuvant ST and forgoing adjuvant RT. While evidence suggests that adjuvant ST alone can reduce the rate of LNB relapse compared to placebo, the role that adjuvant RT may hold in this new era still needs to be defined, especially given the morbidity associated with nodal recurrence [5,6,17,18]. Further, there is a lack of real-world data describing how the recent adoption of adjuvant ST for stage III melanoma has led to changes in clinical practice and patient outcomes [18].

Here, we describe how our institutional practice has changed in response to recent approvals of adjuvant ICIs for stage III melanoma and the associated clinical outcomes, including LNB relapse, relapse-free survival (RFS), and overall survival (OS), in this modern era of melanoma therapy.

## 2. Methods

### 2.1. Data Source and Patient Selection

A single-institution retrospective observational study was conducted with University Health Network research-ethics board approval. Melanoma patients treated with complete lymphadenectomy (CLD) for clinically evident lymph node (LN) disease who presented with high-risk disease between 1 January 2010 and 31 December 2019 were assessed from an institutional cancer registry and clinical records. High-risk features for LNB relapse were defined based on the 2016 CCO guidelines for adjuvant radiation and included any large LNs (≥3 cm), multiple involved LN (≥1 parotid, ≥2 cervical or axillary, ≥3 inguinal or trochlear), and extracapsular extension [13]. Clinically evident disease was defined as lymph nodes that were palpable on physical exam or detected through imaging; this disease could have presented at the time of the initial melanoma diagnosis, or represented a recurrence after prior melanoma treatment with or without sentinel lymph node biopsy (SLNB). Patients with a prior history of a positive SLNB were included if they had not received CLD or adjuvant therapy for microscopic nodal disease. Patients presenting with primary or in-transit disease at the time of LN involvement, or a prior history of primary or in-transit disease, were included only if the cutaneous disease was fully resected prior to or at the time of CLD. Patients were excluded if they presented with distant metastases at the time of LN involvement. Patients with metastases to more than one regional LN basin involved were included so long as all affected LN basins were resected with a lymphadenectomy. In these instances, outcomes were only assessed for affected LN basins that fit the criteria for a high risk of LNB relapse.

Electronic medical records were reviewed to collect demographic, clinical, and pathological data including date of diagnosis, date of CLD, type and duration of adjuvant treatment, and any disease relapse or progression. Scanned pathology reports were consulted, where possible, to collect data describing characteristics of the primary tumor or dissected LNs if surgeries occurred outside of our institution.

### 2.2. Treatments

Specific radiation therapy protocols were selected at the discretion of the treating radiation oncologist. Modern systemic therapies were defined as anti-CTLA-4-, anti-PD-1-, and BRAF/MEK-targeted therapies. All patients that received both adjuvant RT and ST received interferon as ST. In an effort to focus our analyses on modern systemic therapies, these patients were grouped with patients who received adjuvant RT only for analyses. 

### 2.3. Outcomes

This study’s primary outcome was LNB relapse at the site of lymphadenectomy. Secondary outcomes included any disease progression or relapse, overall survival, and the frequency of each adjuvant modality. The duration of time to each event was calculated from the date of CLD to the first event or last follow-up. Patients who experienced disease recurrence or progression within 90 days of CLD were excluded from outcome analysis, with the rationale that recurrence within this timeframe is due to pre-existing disease that was not detected clinically or on imaging at the time of surgery. Patients with pelvic node recurrence following inguinal node lymphadenectomy were classified as having distant recurrence.

### 2.4. Statistical Analysis

Patient characteristics were compared among three treatment groups using chi-squared tests or Fisher’s exact test, as appropriate, for categorical variables and the Kruskal–Wallis test for continuous variables. Comparisons of continuous variables between two time periods were completed using Wilcoxon signed-rank tests. Competing event models were used to assess the cumulative incidence (CIF) of LNB recurrence and the cumulative incidence of any recurrence or progression, where the CIFs were compared using Gray’s test. The competing event was mortality without relevant relapse. Overall survival (OS) was estimated using the Kaplan–Meier method and compared between treatment groups using log-rank tests.

Univariate and multivariable models were applied for LNB recurrence, any recurrence or progression, and overall survival. The Fine–Gray competing risk regression was used for analyses of both LNB recurrence and any recurrence or progression, while the Cox proportional hazards regression was used for analyses of OS. For multivariable models, a backward variable selection method was used and a significance level of *p* <0.05 was required for the covariates, other than the treatment groups, to be retained in the model. The variables included in the full models were those associated with the outcome (*p* < 0.1) identified in the univariate analyses. 

Data management and statistical analyses were performed using SAS software 9.4 (SAS Inc., Cary, NC, USA) and R 4.2.0 (https://www.r-project.org (accessed on 6 December 2022)). The two-sided *p*-value of <0.05 was considered statistically significant. 

## 3. Results

### 3.1. Patient and Treatment Characteristics

A total of 108 eligible patients were included in the analysis; the median age at CLD was 59 years (range 24–92), and 74 (69%) were men (Table 1). A total of 45 (42%) received adjuvant RT only, 22 (20%) received adjuvant ST only, 6 (6%) received both adjuvant RT and ST, and 35 (32%) received no adjuvant therapy. Median follow-up time for patients alive at the last follow-up was 3.7 years and was lower for patients receiving no adjuvant therapy (*p* = 0.009). No significant difference in patient demographics or disease characteristics was observed between treatment groups. Amongst the patients who received adjuvant ST only, 2 (9%) received targeted therapy, and 20 (91%) received ICI. This included patients who were treated as part of trials that, while both arms consisted of ICI therapy, were blinded at the time of analysis. Radiotherapy dosing information was unavailable for nine patients who received their radiotherapy at other hospitals. Regarding the 42 patients for whom RT dose information was available, the median dose was 50 Gy (range 16–70), and the median number of fractions was 25 (range 8–50). One patient did not complete their full course of RT due to adverse effects.

Ninety-one patients did not experience disease recurrence or progression within 90 days of CLD and were included in outcome analyses. Among these patients, only the median size of the largest affected LN was significantly different between treatment groups (*p* = 0.044) (Supplementary Table S1).

### 3.2. Change of Practice

The use of individual adjuvant therapies changed throughout the study period (Table 2). Comparing the patients treated with CLD in the first five years of our period of interest (2010–2014) to those of the latter five years (2015–2019), there was a significant reduction in the use of adjuvant RT and a concurrent increase in the use of adjuvant ST (*p* < 0.001). Patient demographics and disease characteristics did not significantly differ between patients treated before and after 2015 (Supplementary Table S2).

### 3.3. Lymph Node Basin Recurrence

**Figure 1:**
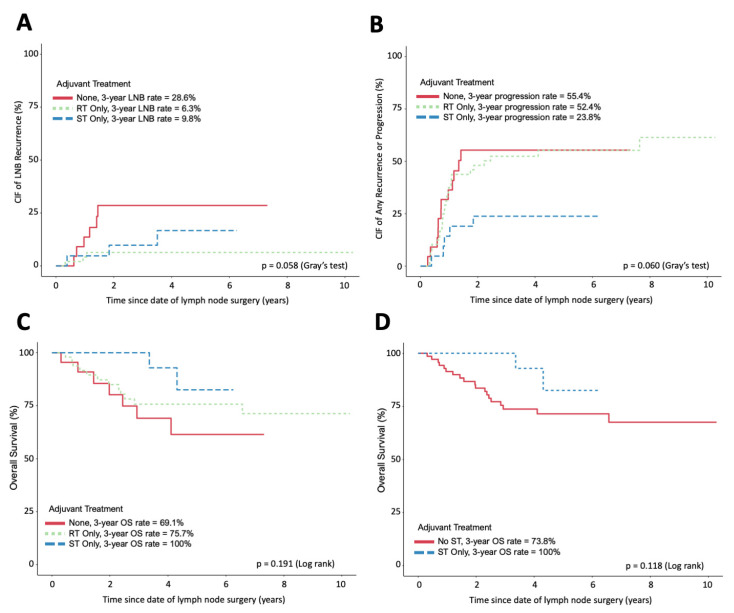
Incidence and survival curves following CLD. (**A**) The cumulative incidence of LNB relapse of each adjuvant treatment group. (**B**) The cumulative incidence of any progression or relapse of each adjuvant treatment group. (**C**) Overall survival of each adjuvant treatment group. (**D**) Overall survival of patients receiving ST compared to those not receiving any ST. (**A**,**B**) Assessed using competing event models. (**C**,**D**) Assessed using Kaplan–Meier and log-rank tests. All figures exclude patients with disease recurrence or progression within 90 days of CLD.

LNB recurrences were a rare event in our study, with only 12 patients (13%) experiencing a recurrence within 3 years. The RT- and ST-only treatment groups each had only three patients experiencing LNB recurrence. The 3-year cumulative incidence of LNB recurrence was similar in patients treated with adjuvant RT only (6.3%) and adjuvant ST only (9.8%), but both were lower than those who received no adjuvant therapy (29%) (Figure 1A). This difference was not statistically significant (*p* = 0.055). In univariate analysis, the time from CLD to LNB recurrence was significantly longer in patients receiving RT as compared to that in patients receiving no treatment (HR *p* = 0.027) (Table 3). However, pairwise comparison revealed no significant difference in time from CLD to LNB recurrence between patients treated with RT versus ST.

A qualitative chart review was also performed to assess clinical outcomes for the five patients in the RT and ST treatment groups who experienced LNB recurrence within 3 years of their LND. The LNB recurrence was treated with salvage LN surgery, the initiation of systemic therapy, or a combination of the two, which resulted in a complete pathological or radiological response within the affected LN basin for four of the five patients for 3 years following treatment of the LNB recurrence (Supplementary Table S3).

### 3.4. Cumulative Incidence of Any Progression or Recurrence and OS

The 3-year cumulative incidence of any disease recurrence or progression was reduced in patients treated with adjuvant ST only (24%) compared to that in patients receiving adjuvant RT (52%) or no adjuvant therapy (55%, *p* = 0.06) (Figure 1B). In a multivariate analysis, the incidence of any disease recurrence or progression was significantly influenced by the ulceration of the primary tumor (HR = 2.11, *p* = 0.003) and the number of affected LNs (HR = 1.05, *p* = 0.006) (Supplementary Tables S4 and S5). OS was not significantly different when comparing the three treatment groups (*p* = 0.191) or in a pooled analysis comparing patients receiving ST to patients receiving no ST (*p* = 0.118) (Figure 1C,D). In multivariate analysis, only ulceration status was found to significantly influence overall survival (HR = 4.22, *p* = 0.003) (Supplementary Tables S6 and S7). 

## 4. Discussion

Our data demonstrate that the advent of systemic therapy has profoundly influenced clinical practice at our institution. Prior to 2015, 68% of patients received adjuvant RT, while only 3% received adjuvant ST excluding interferon. Since 2015, 42% of patients have received ST only in comparison to 21% receiving RT only. This change in practice may be explained, in part, by the enrolment of patients in trials evaluating the efficacy of novel STs such as ICIs and targeted therapies, which were introduced during and after 2015 [5,6,7,16,20]. 

Despite this change in management, LNB recurrence was rare in both treatment groups. We found only six instances of LNB recurrence among all 69 patients treated with either RT or ST. Further, the 3-year cumulative incidence of LNB recurrence was 9.8% for patients receiving ST only and 6.3% for patients receiving RT only, with no significant difference found with competing risk analysis. Although univariate analysis demonstrated that RT was significantly associated with increased time to LNB recurrence compared to no treatment, no significant differences were seen with univariate pairwise testing comparing ST to RT. For patients treated with RT only, our observed 3-year cumulative incidence of LNB recurrence was lower than that observed in the TROG 02.01 trial. For patients treated with ST only, our observed 3-year cumulative incidence of LNB recurrence was also lower than the locoregional recurrence rates reported in previous studies assessing adjuvant ST that excluded adjuvant RT, including 12% for dabrafenib plus trametinib, 17.9% for ipilimumab, 18.7% for nivolumab, and 21.1% for combination ipilimumab plus nivolumab [14,15,16]. An additional retrospective cohort study comparing the use of modern adjuvant ST alone to combined adjuvant ST and RT reported a 3-year incidence of cumulative LNB recurrence as 25.2% and 13.9%, respectively (*p* = 0.36) [21]. Overall, our results indicate that while RT plays a protective role against LNB relapse, the replacement of RT with ST has not necessarily resulted in patients being at higher risk of LNB relapse. Thus, the recommendation of providing adjuvant RT should be challenged given the availability of modern adjuvant ST. Further, our qualitative chart review described several cases where LNB recurrences were successfully managed with surgical re-excision and/or the initiation of ST. Ultimately, the role of adjuvant RT in the modern treatment landscape remains unclear, and the decision to initiate adjuvant RT can be at each clinician’s discretion. Yet, given that local failure rates were similar between patients receiving adjuvant RT vs. adjuvant ST, recommendations to provide adjuvant RT alone should be challenged when modern adjuvant ST is available. Investigations with increased numbers of patients, and the completion of randomized controlled trials are needed to further clarify whether use of ST in place of RT provides equivalent protection against LNB recurrence.

In jurisdictions with ready access to ST for relapsed disease, the use of ST as adjuvant therapy following resection of nodal disease is currently controversial. As evidence suggests ST is associated with increased relapse-free survival without a concordant increase in OS following recurrence, some believe that patients with resected disease could be followed with active surveillance alone, with ST initiation only upon evidence of disease recurrence or distant metastasis [22]. Real-world clinical data from our study demonstrate that patients treated with adjuvant ST had reduced incidence of disease recurrence or progression and improved overall survival compared to all other treatment groups, although these differences did not reach statistical significance.

There are several limitations to our study. First, patients presenting with high-risk nodal disease as defined by the CCO criteria were uncommon at our tertiary, high-volume center; among these patients, LNB recurrences were rare. Together, this made statistical testing for some clinical outcomes difficult, and subsequent multicenter studies may be warranted. Second, for data collection, we used clinic notes to discern the clinical presentation and disease progression of the patients included in this study. However, following the progression of the disease to distant sites, clinic notes often largely discussed the disease burden and management of distant metastasis and neglected a discussion of LNB disease status unless it led to significant morbidity. As a result, our analysis may not have captured all instances of LNB recurrence, especially those that occurred synchronously or after the appearance of stage IV disease. Third, our study did not include patients treated with both adjuvant RT and ST or patients treated with ST in the neoadjuvant setting. Unfortunately, our review of patient records did not yield a sufficient number of patients receiving these treatments to conduct robust analyses. Given the widespread use of modern ST in the adjuvant setting, future research should investigate the role of combining adjuvant ST and RT in preventing LNB recurrence. Fourth, the study could not completely account for other time-dependent practice changes that occurred over the study period. This could include innovations in surgical techniques, improvements in surgical dexterity, or the use of novel technologies in completing surgeries or administering radiotherapy that could have confounded our analysis of patient outcomes. Finally, although there were no significant differences in the clinical and demographic characteristics between treatment groups in our study, given the retrospective nature of our study, there may have been additional factors influencing the choice of treatment that were not accounted for in this study. This further demonstrates the need for future randomized controlled trials assessing the role of adjuvant RT in managing resected stage III melanoma at high risk of nodal recurrence in the era of modern ST. 

## 5. Conclusions

In summary, the approval of adjuvant ST led to a real-world shift away from the use of adjuvant RT in the years following 2015. Our study adds to the growing observational evidence that adjuvant ST reduces the cumulative incidence of any progression or recurrence but it is among the first to suggest that the shift toward adjuvant ST instead of adjuvant RT was not coupled with a significant increase in LNB recurrence among these patients.

## Figures and Tables

**Table 1:** Demographic and clinical characteristics.

	Treatment Group				*p*-Value
	None	RT	ST Only	Total	
	(*n* = 35)	(*n* = 51)	(*n* = 22)	(*n* = 108)	
**Sex**					
Female	13 (37.14%)	16 (31.37%)	5 (22.73%)	34 (31.48%)	0.52
Male	22 (62.86%)	35 (68.63%)	17 (77.27%)	74 (68.52%)	
**Age at CLD**					
Median (Range)	57.6 (38.2–89.8)	59.4 (23.6–83.8)	59.1 (26.7–76.9)	59.1 (23.6–89.8)	0.59
**Primary Breslow’s Depth (mm)**					
Median (Range)	3.0 (0.4–45.0)	2.5 (0.6–35.0)	2.8 (0.4–21.0)	2.6 (0.4–45.0)	0.94
**Melanoma Type**					
Nodular	7 (22.58%)	8 (18.18%)	3 (14.29%)	18 (18.75%)	0.38
Superficial spreading	5 (16.13%)	8 (18.18%)	4 (19.05%)	17 (17.71%)	
Other	12 (38.71%)	12 (27.27%)	3 (14.29%)	27 (28.13%)	
Primary not found	7 (22.58%)	16 (36.36%)	11 (52.38%)	34 (35.42%)	
**Location of Primary**					
Head/neck	13 (37.14%)	13 (25.49%)	5 (22.73%)	31 (28.70%)	0.22
Lower extremity	8 (22.86%)	6 (11.76%)	2 (9.09%)	16 (14.81%)	
Trunk	4 (11.43%)	11 (21.57%)	1 (4.55%)	16 (14.81%)	
Upper extremity	3 (8.57%)	5 (9.80%)	3 (13.64%)	11 (10.19%)	
Primary not found	7 (20.00%)	16 (31.37%)	11 (50.00%)	34 (31.48%)	
**Ulceration of Primary**					
Not Ulcerated	12 (38.71%)	18 (40.91%)	7 (31.82%)	37 (38.14%)	0.23
Ulcerated	12 (38.71%)	10 (22.73%)	4 (18.18%)	26 (26.80%)	
Primary not found	7 (22.58%)	16 (36.36%)	11 (50.00%)	34 (35.05%)	
**Affected LN Location**					
Axilla	11 (31.43%)	21 (41.18%)	9 (40.91%)	41 (37.96%)	0.57
Cervical/Parotid	14 (40.00%)	22 (43.14%)	10 (45.45%)	46 (42.59%)	
Groin/Inguinal	10 (28.57%)	8 (15.69%)	3 (13.64%)	21 (19.44%)	
**Mutation Status**					
BRAF	11 (34.38%)	17 (35.42%)	10 (47.62%)	38 (37.62%)	0.41
Other	6 (18.75%)	8 (16.67%)	6 (28.57%)	20 (19.80%)	
None	15 (46.88%)	23 (47.92%)	5 (23.81%)	43 (42.57%)	
**Affected LN Number**					
Median (Range)	2.0 (1.0–14.0)	2.5 (1.0–32.0)	2.0 (1.0–28.0)	2.0 (1.0–32.0)	0.26
**Largest Affected LN (mm)**					
Median (Range)	28.5 (1.5–90.0)	38.0 (4.0–90.0)	43.0 (12.0–90.0)	35.0 (1.5–90.0)	0.19
**LN Extranodal Extension**					
Absent	18 (69.23%)	28 (65.12%)	10 (52.63%)	56 (63.64%)	0.5
Present	8 (30.77%)	15 (34.88%)	9 (47.37%)	32 (36.36%)	
**ST Type**					
None			0 (0.00%)		
Dabrafenib and Trametinib			2 (9.09%)		
Ipilimumab			2 (9.09%)		
Nivolumab			5 (22.73%)		
Pembrolizumab			5 (22.73%)		
Nivolumab +/− Ipilimumab *			8 (36.36%)		
**Time from CLD to LN Relapse, or Last Follow-Up (years)**					
Median (Range)	1.4 (0.1–7.3)	3.6 (0.2–10.3)	3.6 (0.4–6.3)	2.8 (0.1–10.3)	0.001
**Time from CLD to Death, or Last Follow-Up (years)**					
Median (Range)	2.7 (0.2–7.3)	4.0 (0.5–10.3)	4.0 (1.3–6.3)	3.7 (0.2–10.3)	0.009
**Time from CLD to Any Relapse, or Last Follow-Up (years)**					
Median (Range)	0.6 (0.0–7.3)	1.2 (0.1–10.3)	3.4 (0.1–6.3)	1.2 (0.0–10.3)	0.001

Percentages listed exclude missing values. * These patients were enrolled in the Checkmate 915 trial and were blinded at the time of last follow-up.

**Table 2:** Type of adjuvant therapy used by year of CLD.

Treatment Group	Full Sample (*n* = 108)	2010–2014 (*n* = 60)	2015–2019 (*n* = 48)	*p*-Value
No adjuvant therapy	35 (32%)	17 (28%)	18 (38%)	<0.001
RT Only	51 (47%)	41 (68%)	10 (21%)	
ST Only	22 (20%)	2 (3%)	20 (42%)	

**Table 3:** Univariate analysis for time from complete lymphadenectomy to lymph node basin recurrence based on treatment, as well as patient clinical and demographic characteristics.

Covariate	Level	*n*	Hazard Ratio (95% CI)	HR *p*-Value
**Treatment ^†^**	None	22	Reference	-
	RT	48	0.21 (0.05–0.84)	0.027
	ST only	21	0.47 (0.12–1.81)	0.274
**Sex**	F	29	Reference	-
	M	62	2.46 (0.55–11.07)	0.241
**Melanoma Type**	Superficial spreading	13	Reference	-
	Nodular	16	1.01 (0.22–4.66)	0.994
	Other	23	0.31 (0.05–1.85)	0.198
	Primary not found	30	0.22 (0.04–1.26)	0.090
**Ulceration Status**	Not ulcerated	32	Reference	-
	Ulcerated	21	1.31 (0.35–4.90)	0.681
	Primary not found	30	0.38 (0.08–1.85)	0.23
**Affected LN Location**	Axilla	35	Reference	-
	Cervical/parotid	39	1.66 (0.50–5.50)	0.408
	Groin/inguinal	17	0.52 (0.06–4.54)	0.556
**Mutation Status**	None	34	Reference	-
	BRAF	32	1.05 (0.31–3.58)	0.937
	other	18	0.71 (0.14–3.59)	0.677
**Age at CLD**		91	1.04 (0.98–1.11)	0.202
**Primary Breslow’s Depth**		57	1.07 (0.96–1.20)	0.24
**Affected LN Number**		90	1.04 (0.98–1.11)	0.197
**Largest Affected LN**		82	0.98 (0.95–1.02)	0.283

A competing risk analysis was fitted with the event of interest being 1; death without LN relapse was the competing event [19]. ^†^ On pairwise comparison, there was no difference between ST vs. RT and ST vs. none.

## Data Availability

The data presented in this study are available on request from the corresponding author. The data are not publicly available due to protection of patient information.

## Supplementary Materials

The following supporting information can be downloaded at: https://www.mdpi.com/article/10.3390/cancers15245867/s1, Table S1: Demographic and clinical characteristics among patients who did not relapse within 90 days after CLD; Table S2: Demographic and clinical factors by year of CLD; Table S3: Treatment and outcomes following LNB relapse; Table S4: Univariate survival analysis for time from CLD to any relapse; Table S5: Multivariate survival analysis for time from CLD to any relapse; Table S6: Univariate survival analysis for overall survival (OS); Table S7: Multivariate survival analysis for overall survival (OS).
