# Supplementary material for: The Role of Adjuvant Radiotherapy for the Treatment of Resected High-Risk Stage III Cutaneous Melanoma in the Era of Modern Systemic Therapies
Source: Cancers (Basel). 2023 Dec 16;15(24):5867. doi: 10.3390/cancers15245867 (PMC10741555; doi:10.3390/cancers15245867)
Supplement: Supplementary file 1 [file cancers-15-05867-s001.zip › cancers-2741865-supplementary.pdf]

Supplementary Tables for:

The Role of Adjuvant Radiotherapy for the Treatment of Resected High-Risk Stage III Cutaneous Melanoma in the Era of Modern Systemic Therapies

Seth Kibel [1,2,†], Nathan Kuehne [1,2,†], Mauricio Fernando Ribeiro [2], Thiago P. Muniz [2], Xiang Y. Ye [3], Anna Spreafico [1,2], Samuel D. Saibil [1,2], Alexander Sun [4], David Y. Mak [4], Diana Gray [2], Bailie Jones [2], Philip Wong [4] and Marcus O. Butler [1,2,5,*]

Supplementary Table S1: Demographic and clinical characteristics among patients who did not relapse within 90 days after CLD

Supplementary Table S2: Demographic and clinical factors by year of CLD

Supplementary Figure S3: Treatment and outcomes following LNB relapse

Supplementary Table S4: Univariate survival analysis for time from CLD to any relapse

Supplementary Table S5: Multivariate survival analysis for time from CLD to any relapse

Supplementary Table S6: Univariate survival analysis for overall survival (OS)

Supplementary Table S7: Multivariate survival analysis for overall survival (OS)

Supplementary Table S1: Demographic and clinical characteristics among patients who did not relapse within 90 days after CLD

	Treatment Group				
	**None**	**RT**	**ST only**	**Total**	***P Value***
	**(N = 22)**	**(N = 48)**	**(N = 21)**	**(N = 91)**	
**Sex**					
F	9 (40.91%)	16 (33.33%)	4 (19.05%)	29 (31.87%)	0.29
M	13 (59.09%)	32 (66.67%)	17 (80.95%)	62 (68.13%)	
**Age at CLD**					
Median (Range)	68.7 (39.9 – 89.8)	59.7 (23.6 – 83.8)	59.2 (26.7 – 76.9)	62.6 (23.6 – 89.8)	0.09
**Primary Breslow's Depth (mm)**					
Median (Range)	3.0 (0.4 – 13.0)	2.4 (0.6 – 12.0)	2.5 (0.4 – 21.0)	2.6 (0.4 – 21.0)	0.99
**Melanoma Type**					
Nodular	6 (28.57%)	7 (17.07%)	3 (15.00%)	16 (19.51%)	0.09
Superficial spreading	3 (13.64%)	6 (12.50%)	4 (19.05%)	13 (14.29%)	
Other	9 (42.86%)	12 (29.27%)	2 (10.00%)	23 (28.05%)	
Primary not found	3 (14.29%)	16 (39.02%)	11 (55.00%)	30 (36.59%)	
**Location of Primary**					
Head/neck	10 (45.45%)	12 (25.00%)	4 (19.05%)	26 (28.57%)	0.15
Lower extremity	5 (22.73%)	6 (12.50%)	2 (9.52%)	13 (14.29%)	
Trunk	2 (9.09%)	9 (18.75%)	1 (4.76%)	12 (13.19%)	
Upper extremity	2 (9.09%)	5 (10.42%)	3 (14.29%)	10 (10.99%)	
Primary not found	3 (13.64%)	16 (33.33%)	11 (52.38%)	30 (32.97%)	
**Ulceration of Primary**					
Not Ulcerated	9 (45.00%)	16 (38.10%)	7 (33.33%)	32 (38.55%)	0.13
Ulcerated	8 (40.00%)	10 (23.81%)	3 (14.29%)	21 (25.30%)	
Primary not found	3 (15.00%)	16 (38.10%)	11 (52.38%)	30 (36.14%)	
**Affected LN Location**					
Axilla	6 (27.27%)	20 (41.67%)	9 (42.86%)	35 (38.46%)	0.72
Cervical/parotid	10 (45.45%)	20 (41.67%)	9 (42.86%)	39 (42.86%)	
Groin/Inguinal	6 (27.27%)	8 (16.67%)	3 (14.29%)	17 (18.68%)	
**Mutation Status**					
BRAF	6 (31.58%)	16 (35.56%)	10 (50.00%)	32 (38.10%)	0.29
Other	4 (21.05%)	8 (17.78%)	6 (30.00%)	18 (21.43%)	
None	9 (47.37%)	21 (46.67%)	4 (20.00%)	34 (40.48%)	
**Affected LN Number**					
Median (Range)	2.0 (1.0 – 12.0)	2.0 (1.0 – 32.0)	2.0 (1.0 – 28.0)	2.0 (1.0 – 32.0)	0.19
**Largest Affected LN (mm)**					
Median (Range)	26.0 (12.0 – 90.0)	38.0 (4.0 – 90.0)	44.0 (12.0 – 90.0)	34.5 (4.0 – 90.0)	**0.044**
**LN Extranodal Extension**					
Absent	13 (76.47%)	27 (67.50%)	10 (55.56%)	50 (66.67%)	0.42
Present	4 (23.53%)	13 (32.50%)	8 (44.44%)	25 (33.33%)	
**ST Type**					
None			0 (0.00%)		
Dabrafenib and Trametinib			2 (9.52%)		
Interferon			0 (0.00%)		
Ipilimumab			2 (9.52%)		
Nivolumab			5 (23.81%)		
Pembrolizumab			4 (19.05%)		
Nivolumab +/- Ipilimumab			8 (38.10%)		
**Time from CLD to LN relapse, or last follow-up**					
Median (Range)	1.9 (0.3 – 7.3)	3.9 (0.3 – 10.3)	3.6 (0.4 – 6.3)	3.6 (0.3 – 10.3)	0.07
**Time from CLD to Death, or last follow-up**					
Median (Range)	3.5 (0.3 – 7.3)	4.4 (0.5 – 10.3)	4.2 (1.3 – 6.3)	4.0 (0.3 – 10.3)	0.13
**Time from CLD to any relapse, or last follow-up**					
Median (Range)	1.3 (0.3 – 7.3)	1.8 (0.3 – 10.3)	3.6 (0.4 – 6.3)	2.2 (0.3 – 10.3)	0.26

Supplementary Table S2: Demographic and clinical factors by year of CLD

	Time period		
	2010-2014	***2015-2019***	***P Value***
	(N = 60)	***(N = 48)***	
**Sex**			
F	18 (30.00%)	16 (33.33%)	0.71
M	42 (70.00%)	32 (66.67%)	
**Age at CLD**			
Median (Range)	58.5 (23.6 – 85.9)	59.1 (36.1 – 89.8)	0.48
**Primary Breslow's Depth (mm)**			
Median (Range)	2.4 (0.4 – 45.0)	2.8 (0.4 – 14.0)	0.45
**Melanoma Type**			
Nodular	6 (11.54%)	12 (27.27%)	0.06
Superficial spreading	13 (25.00%)	4 (9.09%)	
Other	17 (32.69%)	10 (22.73%)	
Primary not found	16 (30.77%)	18 (40.91%)	
**Location of Primary**			
Head/neck	22 (36.67%)	9 (18.75%)	0.33
Lower extremity	8 (13.33%)	8 (16.67%)	
Trunk	9 (15.00%)	7 (14.58%)	
Upper extremity	5 (8.33%)	6 (12.50%)	
Primary not found	16 (26.67%)	18 (37.50%)	
**Ulceration of Primary**			
Not Ulcerated	22 (43.14%)	15 (32.61%)	0.55
Ulcerated	13 (25.49%)	13 (28.26%)	
Primary not found	16 (31.37%)	18 (39.13%)	
**Affected LN Location**			
Axilla	21 (35.00%)	20 (41.67%)	0.18
Cervical/parotid	30 (50.00%)	16 (33.33%)	
Groin/Inguinal	9 (15.00%)	12 (25.00%)	
**Mutation group**			
BRAF	22 (40.00%)	16 (34.78%)	0.15
None	26 (47.27%)	17 (36.96%)	
Other	7 (12.73%)	13 (28.26%)	
**Affected LN Number**			
Median (Range)	2.0 (1.0 – 32.0)	2.0 (1.0 – 28.0)	0.48
**Largest Affected LN (mm)**			
Median (Range)	35.0 (4.0 – 90.0)	35.5 (1.5 – 90.0)	0.52
**LN Extranodal Extension**			
Absent	35 (70.00%)	21 (55.26%)	0.15
Present	15 (30.00%)	17 (44.74%)	

Supplementary Table S3: Treatment and outcomes following LNB relapse

Adjuvant Treatment Group	Relation to progression to stage IV	Location of Relapse	Time from CLD to recurrence (days)	Treatment	Outcome
ST only (ipilimumab)	LNBr first	Right neck	140	Repeat lymph node dissection, repeat ipilimumab therapy	Two years disease free until distant progression as well as right cervical LN recurrence
ST only (nivolumab)	LNBr only	Right axilla	673	Repeat lymph node dissection, start targeted therapy	No subsequent disease progression
RT only	Synchronously with distant progression	Left neck	342	Initiation of ipilimumab	Complete response to ipilimumab
RT only	LNBr first	Right parotid	390	Surgical re-excision	One year disease free until distant progression. No subsequent local or regional recurrence.
RT only	Synchronously with distant progression	Left axilla	117	Treatment with vemurafenib	Complete response to vemurafenib

Supplementary Table S4: Univariate survival analysis for time from CLD to any relapse

Covariate	Level	N	Hazard Ratio (95% CI)	HR P-value
**Treatment**	None	22	Reference	-
	RT	48	0.93 (0.47-1.84)	0.83
	ST only	21	0.33 (0.12-0.95)	**0.039**
**Sex**	F	29	Reference	-
	M	62	0.89 (0.48-1.66)	0.713
**Melanoma Type**	Superficial spreading	13	Reference	-
	Nodular	16	2.18 (0.82-5.85)	0.120
	Other	23	1.64 (0.64-4.20)	0.298
	Primary not found	30	0.59 (0.22-1.59)	0.297
**Ulceration Status**	Not Ulcerated	32	Reference	-
	Ulcerated	21	2.07 (1.03-4.17)	**0.044**
	Primary not found	30	0.50 (0.22-1.11)	0.088
**Affected LN Location**	Axilla	35	Reference	-
	Cervical/parotid	39	1.29 (0.66-2.51)	0.462
	Groin/Inguinal	17	1.68 (0.78-3.63)	0.187
**Mutation Status**	None	34	Reference	-
	BRAF	32	0.81 (0.43-1.52)	0.506
	other	18	0.48 (0.19-1.24)	0.129
**Age at CLD**		91	1.03 (1.00-1.06)	0.063

**Primary Breslow's Depth (mm)**		57	1.06 (0.97-1.17)	0.194
**Affected LN Number**		90	1.05 (1.02-1.07)	**<0.001**
**Largest Affected LN (mm)**		82	0.99 (0.98-1.01)	0.394

Supplementary Table S5: Multivariable survival analysis for time from CLD to any relapse

Covariate	Level	N	Hazard Ratio (95% CI)	HR P-value	Type3 P-value
**Treatment**	None	20	Reference	-	
	RT	41	0.99 (0.46-2.10)	0.97	0.111
	ST only	21	0.39 (0.13-1.12)	0.080	
**Ulceration Status**	Not Ulcerated	32	Reference	-	
	Ulcerated	21	2.09 (1.03-4.23)	**0.042**	**0.009**
	Primary not found	29	0.59 (0.27-1.31)	0.196	
**Affected LN Number**		82	1.05 (1.02-1.09)	**0.005**	**0.005**

Number of observations used = 82. Backward selection with an alpha level of removal of 0.05 was used. The following variables were forced in the model: Treatment. The following variables were removed from the model: Age at CLD. In pairwise comparisons, the HR (ST only vs RT) was significantly less 1 (p = 0.032), with no significant difference between ST only and None groups.

Supplementary Table S6: Univariate survival analysis for overall survival (OS)

Covariate	Level	N	Hazard Ratio (95% CI)	HR P-value	Log-rank P-value
**Treatment**	None	22	Reference	-	0.191
	RT	48	0.67 (0.26-1.71)	0.398	
	ST only	21	0.25 (0.05-1.22)	0.086	
**Sex**	F	29	Reference	-	0.976
	M	62	0.99 (0.40-2.45)	0.976	
**Melanoma Type**	Superficial spreading	13	Reference	-	**0.055**
	Nodular	16	2.54 (0.65-9.835	0.179	
	Other	23	1.30 (0.34-5.06)	0.700	
	Primary not found	30	0.52 (0.12-2.31)	0.387	
**Ulceration Status**	Not Ulcerated	32	Reference	-	**<0.001**
	Ulcerated	21	4.27 (1.57-11.61)	**0.004**	
	Primary not found	30	0.68 (0.19-2.43)	0.557	
**Affected LN Location**	Axilla	35	Reference	-	0.067
	Cervical/parotid	39	2.63 (0.84-8.29)	0.098	
	Groin/Inguinal	17	4.12 (1.16-14.67)	**0.029**	
**Mutation Status**	None	34	Reference	-	0.507
	BRAF	32	1.61 (0.61-4.26)	0.34	
	Other	18	0.92 (0.27-3.15)	0.894	
**Age at CLD**		91	1.02 (0.99-1.05)	0.194	-
**Primary Breslow's Depth (mm)**		57	1.15 (1.05-1.25)	**0.002**	-
**Affected LN Number**		90	1.02 (0.95-1.10)	0.516	-
**Largest Affected LN (mm)**		82	0.99 (0.97-1.01)	0.543	-

Supplementary Table S7: Multivariate survival analysis for overall survival (OS)

Covariate	Level	N	Hazard Ratio (95% CI)	HR P-value	Type3 P-value
**Treatment**	None	20	Reference	-	0.386
	RT	42	0.96 (0.36-2.54)	0.927	
	ST only	21	0.34 (0.07-1.72)	0.194	
**Ulceration Status**	Not Ulcerated	32	Reference	**-**	**0.003**
	Ulcerated	21	4.22 (1.54-11.62)	**0.005**	
	Primary not found	30	0.81 (0.22-2.91)	0.744	

Number of observations used = 83. This final model was derived by backward variables selection procedure with variable stay criterion of p<0.05, where treatment was forced in the model. The variables included in the full model were treatment, Melanoma type, Ulceration status, Affected LN location. Primary Breslow's Depth was significantly associated with the OS in the subpopulation of patients where Primary Breslow's Depth was assessable.
